# Updating constructions: additive effects of prior and current experience during sentence production

**DOI:** 10.1515/cog-2022-0020

**Published:** 2023-10-03

**Authors:** Malathi Thothathiri, Natalia Levshina

**Affiliations:** Department of Speech, Language and Hearing Sciences, The George Washington University, Washington, USA; Max Planck Institute for Psycholinguistics, Nijmegen, Netherlands

**Keywords:** dative constructions, verb bias, error-based learning, exemplar theory, Bayesian regression

## Abstract

While much earlier work has indicated that prior verb bias from lifelong language experience influences language processing, recent findings highlight the fact that verb biases induced during lab-based exposure sessions also influence processing. We investigated the nature of updating, i.e., how prior and current experience might interact in guiding subsequent sentence production. Participants underwent a short training session where we manipulated the bias of known English dative verbs. The prior bias of each verb for the double-object (DO) versus the prepositional-object (PO) dative was estimated using a corpus. Current verb bias was counterbalanced and controlled experimentally. Bayesian mixed-effects logistic models of participants’ responses (DO or PO) during subsequent free-choice production showed that both the prior and current verb biases affected speakers’ construction choice. These effects were additive and not interactive, contrary to the prediction from error-based learning models. Semantic similarity to other verbs and their experimentally manipulated biases influenced sentence production, consistent with item-based analogy and exemplar theory. These results shed light on the potential mechanisms underlying language updating and the adaptation of sentence production to ongoing experience.

## 1 Aims of the study

Language learning and use are sensitive to statistical frequency at different levels, including phonotactics, lexical access, and sentence formulation. Studies with infants demonstrate that their brains implicitly track and analyze the distributional properties of language input (see Romberg and Saffran 2010 for a summary). Adults learning an artificial language in laboratory settings show similar sensitivity to input statistics (e.g., Perek and Goldberg 2017; Thothathiri and Rattinger 2016; Wonnacott et al. 2008). Adults also demonstrate implicit knowledge of the distributional statistics of their native language (Ellis 2002). Sensitivity to statistical properties is not just a feature of the phase in which language is acquired, however. A key premise of theories that emphasize the role of language experience in language use, including usage-based construction grammar, is that language users continually update their linguistic representations (Ellis 2002). In this study, we investigated the mechanisms of native-language-updating within the context of verb bias and sentence production.

A rich body of evidence shows that sentence processing is sensitive to verb bias, the statistical preference of a given verb for some structures over others. Language users seem to implicitly know how often a verb appears in different structures (e.g., active vs. passive) and the types of sentential arguments it takes (see, e.g., Ellis 2002 for a summary). Recent findings additionally show that statistical properties manipulated during lab-based exposure sessions influence adults’ subsequent sentence comprehension and production (Fraundorf and Jaeger 2016; Kamide 2012; Kroczek and Gunter 2017; Ryskin et al. 2017; Thothathiri et al. 2017; Thothathiri and Braiuca 2021). For example, Ryskin and colleagues showed that verbs’ biases for appearing in different structures as controlled by the experimenters influenced participants’ subsequent comprehension. Kroczek and Gunter (2017) found that a single session of exposure to different speakers with different word order preferences influenced listeners’ expectations during the comprehension of utterances by those speakers.

For sentence production, corpus analyses show that different verbs, even near-synonymous ones, can have different structural preferences. For example, *give* tends to appear more often in double-object datives (DO e.g., *Laila gave Bob the money*) than in prepositional-object datives (PO e.g., *Laila gave the money to Bob*) (Gries 2005). In contrast, *donate* is similar in meaning to *give* but is highly biased towards PO datives (Gropen et al. 1989). In fact, usage of this verb in DO datives is unacceptable for most speakers of American English (*Laila donated B. the money). Experimental studies of sentence production further show that verbs’ statistical tendencies, as measured via corpus analysis, can influence how speakers choose to use those verbs (Stallings et al. 1998). For example, Stallings et al. (1998) found that a verb’s disposition to take or not take sentential complements (e.g., *John revealed that he was moving* vs. *John transferred that he was moving) had an effect on how often speakers were willing to shift heavy noun-phrases away from that verb during language production. In sum, verb bias can influence sentence production as well as comprehension.

But, can speakers flexibly adapt sentence production if they experience different verb biases than in the past, as found in sentence comprehension studies (Kroczek and Gunter 2017; Ryskin et al. 2017)? Two recent studies suggest that they can (Thothathiri et al. 2017; Thothathiri and Braiuca 2021). In Thothathiri et al. (2017), the experimenters pseudorandomly assigned different dative verbs to different bias conditions such that participants heard some verbs exclusively in DO datives (DO-only), others exclusively in PO datives (PO-only), and yet others equally in both (Equal-DO-PO). They tested participants’ subsequent production with these verbs and found that the likelihood of using a verb in a DO sentence patterned according to lab-manipulated verb bias (DO-only > Equal-DO-PO > PO-only). Speakers were most likely to produce DO with verbs that had been heard only in that structure during lab-based exposure and least likely to do so with verbs that had been heard only in the competing PO, with Equal-DO-PO verbs in between. Thothathiri and Braiuca (2021: Experiment 1) found a similar effect. Thus, short exposure sessions where verbs in the participants’ native language appeared in different structures according to experimentally designed manipulations can influence speakers’ subsequent use of those verbs, indicating an ability to adapt to the statistical properties of ongoing language input.

What kinds of mechanisms could support such adaptation or updating? Error-based learning theories are the predominant models in the domain of verb-structure links (Chang et al. 2006; Jaeger and Snider 2013). According to these theories, the sentence processing system continuously predicts upcoming linguistic material at different levels (phonemes, words, sentence structures).1Error-based learning is closely linked to statistical learning because the predictions arise from tracking statistical regularities in the input (Christiansen 2019; Peter and Rowland 2019). If the actual material differs from the predicted material, connection weights between representations are adjusted. Crucially, this adjustment (i.e., learning) is expected to be larger if the error or discrepancy between the predicted and actual material is larger. For example, in the case of syntactic alternations, a strongly PO-biased verb in a DO structure is more discrepant than a weakly PO-biased verb in a DO structure and is therefore expected to lead to more learning. Specifically, the weights connecting verbs to dative structures is hypothesized to be adjusted to increase the weight for DO and decrease the weight for PO, and more so when the listener encounters a strongly PO-biased than a weakly PO-biased verb in the DO structure because the former is more surprising and should lead to a larger prediction error.

Evidence for error-based learning comes from “surprisal” effects in syntactic priming as well as training paradigms. For example, Bernolet and Hartsuiker (2010) found that participants were more likely to use a DO structure if they had just heard a prime sentence containing the DO structure. However, this main effect of syntactic priming was modulated by the prime verb’s bias—a more “surprising” prime sentence that went against the verb’s typical structural preference influenced participants’ subsequent production more. Similarly, in a training study with children and adults, Lin and Fisher (2017) found that unexpected verb-structure combinations heard during training created larger effects on later sentence production. Such findings are consistent with error-based learning because speakers’ production choices appear to be sensitive to the mismatch between what they expect based on prior verb bias and what they actually hear.

It is worth clarifying here that prediction and sensitivity to prediction error are also a part of error-driven learning theories (see e.g., Ramscar et al. 2013), where the focus is typically on determining which cues are predictive of different outcomes. For example, in the context of the experimental paradigm used here, error-driven learning theories could be used to model whether learners come to rely on individual verbs or broader event semantics for predicting the sentence structure heard during training (see Thothathiri and Braiuca 2021 and Thothathiri and Rattinger (2016) for related discussions about cue validity). The specific training statistics for the experiment analyzed here (Thothathiri et al. 2017) clearly favors individual verbs as predictive cues because 8/10 verbs appeared consistently in one structure (cue validity = 0.9, see Thothathiri and Braiuca 2021). Event semantics, by contrast, was unpredictive of structure because any given transfer event was equally likely to be described by DO or PO (cue validity = 0.5). Thus, the finding that participants relied on the verb cue and learned verb bias (Thothathiri et al. 2017) is consistent with error-driven learning. The question in this paper is at a different nested level—given that the verb was relevant for predicting structure, were participants specifically relying on using the prior bias of the verb and the mismatch/error between what the prior bias predicted and what the current structure in the experiment was, in order to update verb-structure links? Error-based learning is the term used in the literature for models at this particular level (see e.g., Fazekas et al. 2020).

Thothathiri et al. (2017) found that participants adapted to the experimentally manipulated biases of English dative verbs, as shown by an effect on their utterance choices. In this paper, we analyzed the same dataset to investigate whether this updating of links between verbs and structures showed a key signature of error-based learning, namely sensitivity to the match or mismatch between prior bias and current bias. Did participants adapt more when the input was more surprising from the perspective of previous language experience? To answer this question, we estimated prior verb bias using corpus analysis and used Bayesian modeling to evaluate whether prior and current verb bias showed an interaction. An interaction effect is expected under error-based learning because the mechanism is contingent on setting up expectations based on prior bias and then updating according to how the current experience aligns with those expectations. Alternative paths to learning and updating verb biases are possible. For example, tracking usage statistics without regard to match with prior bias would lead to updating of verb-structure links but this updating would show additive rather than interactive effects of prior and current bias.

To preview our results, we did not find evidence in favor of error-based learning. Therefore, we considered post-hoc whether Exemplar Theory, sans error-based learning assumptions, might be a potential alternative explanation for how participants represented and used input statistics in our paradigm. Accordingly, as a secondary goal we tested whether semantic similarity between verbs influenced the use of DO and PO, which would be expected if production was guided by item-based analogy from exemplars (Bybee 2013; Nosofsky 1988). Previous studies have provided support for a role for semantic similarity in how constructions are used with different lexical items. A study of Spanish verbs of ‘becoming’ by Bybee and Eddington (2006) showed that the acceptability ratings of sentences with different copula verbs were higher for high-frequency combinations of the copula verbs and adjectives (e.g., *ponerse nervioso* ‘get nervous’) than for rare combinations (e.g., *ponerse viejo* ‘become old’). Importantly, the ratings were also high for those adjectives that were used infrequently with the verbs but were semantically similar to the adjectives occurring in the high-frequency combinations. This effect is consistent with Exemplar Theory, according to which categories are structured depending on the frequency and similarity of their exemplars (Nosofsky 1988). More recently, Yi et al. (2019) showed that high distributional semantic similarity to the verb *give*, which is the most frequent verb in the DO construction, increases the likelihood of an alternating verb (e.g., *to hand*) occurring in DO versus PO. Numerous other studies suggest that semantic similarity of novel items to already existing exemplars affects constructional productivity (e.g., Barðdal 2008; Perek 2016; Suttle and Goldberg 2011). Together, such effects demonstrate that language learning and use strongly depend on exemplar representations of constructions in memory and similarities between those representations (Bybee 2013).

In the present study, we used English dative verbs for which the participants had presumably learned the semantic similarity structure already from life-long experience. During the experiment, we manipulated the verb bias of different verbs. In the training phase, participants heard 4 verbs in DO-only, 4 verbs in PO-only, and 2 verbs in Equal-DO-PO. Which verb was in which bias condition was counterbalanced across lists. Thus, any given verb in any given list could be semantically similar to one or more verbs in the DO-only, PO-only, or Equal-DO-PO conditions. We hypothesized that whether a verb was more semantically similar to verbs that appeared in DO versus PO within the experiment could influence how that verb was used. Specifically, we predicted that if a verb shared greater semantic similarity with DO-only than PO-only verbs, that would increase the likelihood of producing a DO construction. Conversely, if it was more similar to PO-only than DO-only verbs, that would increase the likelihood of a PO construction. In sum, we tested whether the semantic similarity structure between dative verbs–learned from life-long experience–interacted with verb bias manipulated during the experiment. While prior evidence has already shown that semantic similarity affects how verbs are used in different constructions, the proposed analysis would clarify if semantic similarity is integrated with *updates* to verb biases (i.e., whether it influences production upon adaptation to novel statistical information).

The rest of the paper is structured as follows. In Section 2, we test the prediction based on error-based learning. In Section 3, we test the semantic similarity prediction based on Exemplar Theory. Finally, Section 4 discusses conclusions and future directions.

## 2 Testing the error-based learning prediction

Our primary analyses tested whether there was an interaction between prior and current verb bias on construction choice, as predicted by error-based learning theories.

### 2.1 Methods

#### 2.1.1 Experimental data set

We analyzed the dataset from Thothathiri et al. (2017), which included sentence production data from 88 right-handed native English speakers taking part in an experiment. During the experiment, participants underwent a short training session where they watched videos of transfer actions and heard an accompanying DO or PO sentence (e.g., *Kate gave the tiger the cup*). Each video showed a human hand transferring an object to a puppet animal. Six objects (apple, candle, cup, flower, fork, and hat) and six animals (donkey, giraffe, lion, monkey, tiger, zebra) appeared 20 times each. Ten verbs (bring, give, mail, offer, pass, roll, show, slide, throw, toss) were heard 12 times each for a total of 120 training trials. Of the ten verbs, four were heard only in DO sentences (hereafter, DO-only verbs), four only in PO sentences (PO-only), and two equally in both (Equal-DO-PO). The assignment of verbs to bias conditions was counterbalanced across lists. Each participant was randomly assigned to a list. Trial order was pseudorandomized such that verbs did not repeat consecutively, and there were no more than three trials from the same verb bias condition in a row. Participants advanced from one trial to the next manually by pressing a key.

Subsequent to training, participants watched new videos of transfer actions and were asked to describe what they saw on their own. These test videos contained new objects (feather, key, knife, mitten, napkin, ring) and animals (bear, cat, cow, dog, frog, pig) compared to training. Thus, participants had to generate sentences and could not simply reproduce memorized sentences from before. On each test trial, participants saw a written verb followed by the video. Then they saw a slide with a green circle, which was their cue to speak. They were given up to 4 s to produce a verbal response. When done, they pressed a key to advance to the next trial. Each verb appeared four times and each animal/object appeared six or seven times. A total of 40 test trials were presented in two blocks of 20. Trial order was pseudorandomized such that consecutive trials did not contain the same verb or nouns. There were no more than two trials from the same verb bias condition in a row.

We transcribed and coded participants’ descriptions of the test videos. Responses with the correct verb, correct nouns (or near synonyms e.g., glove for mitten), and a complete dative structure including an agent, a recipient and a theme were coded as DO/PO. Non-dative structures and errors (e.g., repeats, restarts, incorrect verb, incorrect noun) were coded as Other. Please see Thothathiri et al. (2017) for additional details.

#### 2.1.2 Corpus analysis: estimating prior verb bias

We extracted frequency data from COCA, the Corpus of Contemporary American English (Davies 2008–) for the verbs used in the experiment. The purpose was to estimate the prior biases of the verbs towards DO or PO. We chose COCA because it is a very large corpus (500 million words available in our local copy) representing diverse speech genres. A large and diverse corpus was a necessity due to the rare occurrence of some of the verbs in the dative alternation. For the corpus analysis, we first extracted all instances of the verbs together with their context. All morphological forms with verb tags (“v**”) were counted in (e.g., *give*, *gives*, *gave*, *given* and *giving*). Next, we annotated the sentences syntactically with the help of the package *spacy_udpipe* in Python,2https://spacy.io/universe/project/spacy-udpipe (last access 04.11.2021). which provides annotation in the Universal Dependencies style (Zeman et al. 2021) based on pre-trained language models for different languages, including English. This enabled us to collect information about the verbs’ occurrences as Verb slot fillers in the DO and PO constructions. A syntactic configuration was counted as an instance of DO if the verb had simultaneously a direct object (represented by a Universal Dependency “obj”) and indirect object (“iobj”), and as an instance of PO if the verb had a direct object and an oblique phrase (“obl”) with the preposition *to*. Finally, for every verb, we computed the proportion of its use in DO divided by the sum of its uses in DO and PO.

The counts and proportions are shown in Table 1. The frequencies show that *give* and *show* have a DO bias, while all verbs of caused motion have a PO bias. The relative frequencies of DO and PO in our data for the verbs *bring*, *give*, *pass* and *show* are similar to the ones in Gries and Stefanowitsch (2004), who used the ICE-GB corpus. The verb *offer* is, however, DO-biased in the ICE-GB corpus (74 %), whereas in our data it shows a weak bias towards the PO dative (56.3 %). The verb *post*, which is more frequently used in British English, has a PO bias in ICE-GB, similar to the verb *mail* in our COCA data.

**Table 1:** Counts and proportions of the dative constructions in COCA.

Verb	Count (Proportion as a percentage)	
	DO	PO
Bring	5,373 (14.8 %)	30,848 (85.2 %)
Give	138,232 (83.6 %)	27,022 (16.4 %)
Mail	118 (23 %)	394 (77 %)
Offer	4,279 (43.7 %)	5,504 (56.3 %)
Pass	343 (10.2 %)	3,017 (89.8 %)
Roll	33 (8.4 %)	362 (91.6 %)
Show	11,308 (73.6 %)	4,059 (26.4 %)
Slide	11 (6.8 %)	180 (93.2 %)
Toss	201 (24.3 %)	627 (75.7 %)
Throw	633 (29 %)	1,547 (71 %)

#### 2.1.3 Bayesian modeling: investigating interaction between prior and current verb bias

To investigate the mechanisms underlying updating, we used Bayesian modeling to test whether there was an interaction between prior verb bias (from the corpus) and current verb bias (from the experiment). Such an interaction is expected under error-based theories, which predict that a greater mismatch between prior and current verb bias should lead to stronger learning (i.e., more alignment with the current bias).

The model contained the following variables. *ResponseType* (dependent variable) represented the choice of dative construction in the test phase. The values were “DO” (reference level) and “PO”. The key predictor variables were *CurrentBias* (DO-only, Equal-DO-PO, PO-only. DO-only was the reference level) and *PriorBias* (numerical value corresponding to the proportion of PO from the corpus analysis. See Table 1). PriorBias was also tested in the form of log-odds of PO relative to DO in the corpus. The results were nearly identical (see Supplementary Material SM1).

We included four other predictor variables that could potentially influence language production. The first was the length of the noun arguments. Longer noun phrases are more likely to be shifted to the end of the sentence (e.g., Bresnan et al. 2007). In the present study, the only length variation was in the object argument. All recipient arguments were single syllable words. Half the objects were 2 syllables and half were 1 syllable. We coded this as a dichotomous variable, indicating whether the object was longer than the recipient (*ObjectLonger* = “No” (reference level) or “Yes”). The second additional predictor was *PreviousResponse*, or the dative construction (DO or PO) produced by the participant on the previous trial. This allowed us to capture possible syntactic priming effects (Bock 1986; Pickering and Branigan 1999). The final predictor variables were *Block* (first or second) and *Trial* (1–20 within each block), which could capture any effects of time (such as fatigue). Random variables included *Subject, Verb*, *EventVideoClip* (each video clip item), and *List* (each participant was assigned to one of five counterbalanced lists). Including *List* as a random variable allowed us to generalize to all possible random lists. *EventVideoClip* was nested under *Verb.*

The data comprised a total of 3,189 observations. We excluded trials with missing *PreviousResponse* values (the first trial, or if the previous trial had a response other than DO or PO), resulting in a final dataset of 3,035 observations. We fitted this dataset to a series of Bayesian mixed-effects logistic regression models using the package brms (Bürkner 2018). The models were fitted with weakly informative Cauchy priors (0, 2.5) for the fixed effects estimates and half-Cauchy priors for the standard deviations of the random effects. These priors were chosen because they constrained the estimates (log-odds) within realistic boundaries acceptable for logistic regression but did not influence the direction of effects. We first created models based on four Markov chains with 2,000 iterations in each (1,000 after the warm-up period), in order to test that the chains converged (they did). In the final model reported below, there was one large Markov chain, which included 10,000 steps, with the warm-up period of 2,000 iterations. The large chain allowed us to obtain more precise estimates of the regression coefficients derived from posterior distributions. The delta parameter of 0.99 was used in order to avoid divergent transitions. All R-hat values were 1.00, which shows that the chain mixed well.

The model contained all random intercepts. Because of the high number of potential random slopes (maximally, six fixed effects by four grouping factors would result in 24 random slopes), the parsimonious approach was chosen over the maximal approach, following the argumentation in Bates et al. (2018) and Matuschek et al. (2017). Random slopes were tested with the help of the WAIC criterion. A random slope was selected if the model including it had higher predictive power than the model without it, and the absolute difference was at least twice as high as the standard error of that difference. This procedure allowed us to identify the random slopes of *PreviousResponse*, *Block* and *Trial* for *Subject*. We tested an interaction term between *Trial* and *PreviousResponse* as a random slope for *Subject* because there was an interaction between the fixed effects, but we did not include it in the final models (see below; see also Supplementary Material SM2). In addition, we fitted maximum likelihood models and performed likelihood ratio tests in order to determine the best random effects structure. The results of the two approaches converged.

The model with the random effects and the fixed effects only was compared with models containing all possible pairwise interactions between the fixed effects. Interactions were accepted if their 95 % credible intervals did not include zero.3We used this filtering approach solely to keep the model interpretable and focus on theoretically important factors and their interactions. Thus, we applied the filtering to non-theoretically-motivated effects only. Factors and interactions for which we had a-priori principled expectations were retained and reported regardless of the findings. The interaction between *Trial* and *PreviousResponse* was found to correspond to meet this criterion, although it was quite weak. We did not discover any non-linear effects, using the GAM smooths (Wood 2017), so no transformation was needed.

The structure of the final model was as follows:

(1) ResponseType ∼ CurrentBias + PriorBias + ObjectLonger + PreviousResponse + Block + Trial + Trial:PreviousResponse + (1 + PreviousResponse + Block + Trial |SubjectID) + (1|Verb/EventVideoClip) + (1|List)

The goodness-of-fit statistics indicated that the fit was good. The Bayesian C-index (the index of concordance, or the area under the ROC curve) mean was 0.849, with the 95 % credible interval between 0.842 and 0.855. The C-index is considered acceptable if it is above 0.7 and excellent if it is above 0.8 (Hosmer and Lemeshow 2000: 162).

### 2.2 Results

Table 2 displays the mean posteriors of the fixed effects and their 95 % credible intervals. The numbers represent log-odds for the intercept and log-odds ratios for the predictors. The table also contains the probabilities of positive values of the coefficients (computed as the proportion of positive posteriors of the coefficients in the posterior samples). Positive log-odds mean that the variable or its value increases the chances of PO and decreases the chances of DO. Negative numbers indicate that the variable or value increases the chances of DO and decreases the chances of PO.

**Table 2:** Mean posteriors and their 95 % credible intervals of the fixed effects in Bayesian GLMM. The posteriors represent log-odds in favor of PO versus DO.

Regression term	Mean posterior (log-odds)	Lower 95 % CI	Upper 95 % CI	Posterior probability *b* > 0
Intercept	−1.08	−2.21	0.10	3.36 %
Trial	0.02	−0.01	0.06	91.75 %
Block = 2 (vs. 1)	−0.20	−0.59	0.17	14.38 %
**PreviousResponse = PO (vs. DO)**	**0.67**	**0.14**	**1.19**	**99.46 %**
CurrentBias = Equal-DO-PO (vs. DO-only)	0.18	−0.07	0.43	91.45 %
**CurrentBias = PO-only (vs. DO-only)**	**0.22**	**0.02**	**0.43**	**98.55 %**
**PriorBias in favor of PO**	**1.66**	**0.54**	**2.70**	**99.6 %**
ObjectLonger = Yes (vs. No)	0.08	−0.16	0.32	75.75 %
**Trial: PreviousResponse = PO (vs. DO)**	−**0.04**	−**0.08**	−**0.01**	**0.86 %**

Effects where the 95% credible interval does not span zero are highlighted in bold.

**Figure 1:**
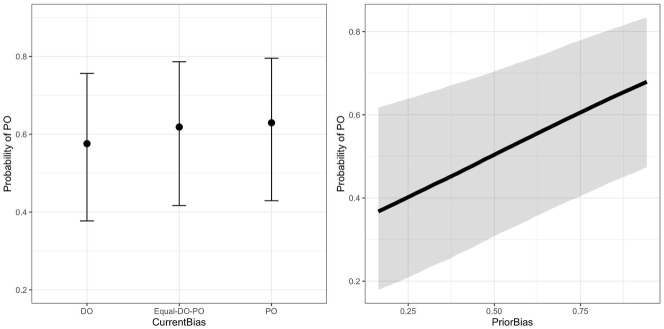
Partial effects of two types of verb bias on the probability of PO. Left: The effect of *CurrentBias* on the probability of PO. Right: The effect of *PriorBias* towards PO on the probability of PO. As current or prior bias towards PO increases, the probability of producing a PO increases. The error bars and the ribbon represent the 95 % CIs based on posterior draws.

There was an effect of both *CurrentBias* and *PriorBias*. For *CurrentBias*, the chances of the speaker producing a PO utterance were higher for verbs with PO-only bias compared to verbs with DO-only bias (the effect was in the same direction but weaker for the comparison between Equal-DO-PO and DO-only). For *PriorBias*, verbs with prior PO bias increased the likelihood of a PO response compared to verbs with prior PO bias. Thus, both variables influenced production to be more aligned with the verb’s bias (current or prior). Figure 1 is a partial effects plot, which represents the effects of each variable on the probability of PO while keeping the other predictors constant (the mean values for all numeric variables and reference levels for all categorical variables).

**Figure 2:**
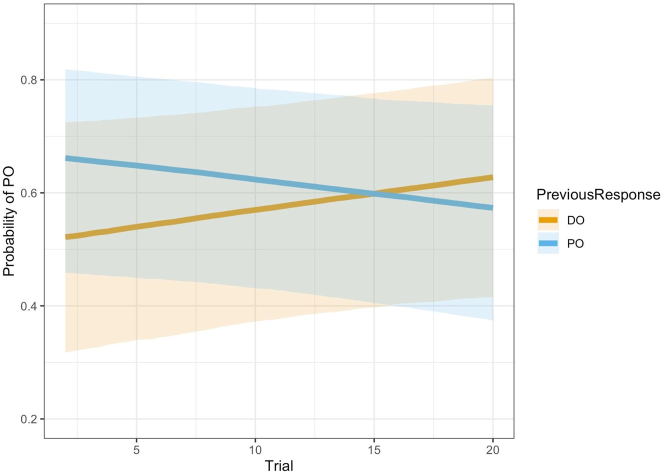
Interaction between *PreviousResponse* and *Trial*. Participants were more likely to produce a PO if the previous response was a PO during earlier but not later trials.

Additionally, we found a main effect of *PreviousResponse* and an interaction between *Trial* and *PreviousResponse*. There was a syntactic self-priming effect such that speakers were more likely to produce a PO if they had produced a PO on the previous trial. This effect, however, decreased with trial number, meaning that the effect was present at the beginning but not at the end of a block. The interaction is displayed in Figure 2. Over time, the differences between previous DO and PO disappear. At the end, there is even a slightly higher chance of PO in the target sentence if the previous sentence contained a DO than if it contained a PO.

There was no noteworthy effect of *ObjectLonger* (relative length of Object and Recipient). This could be due to the fact that the differences were very small (one syllable only).

**Figure 3:**
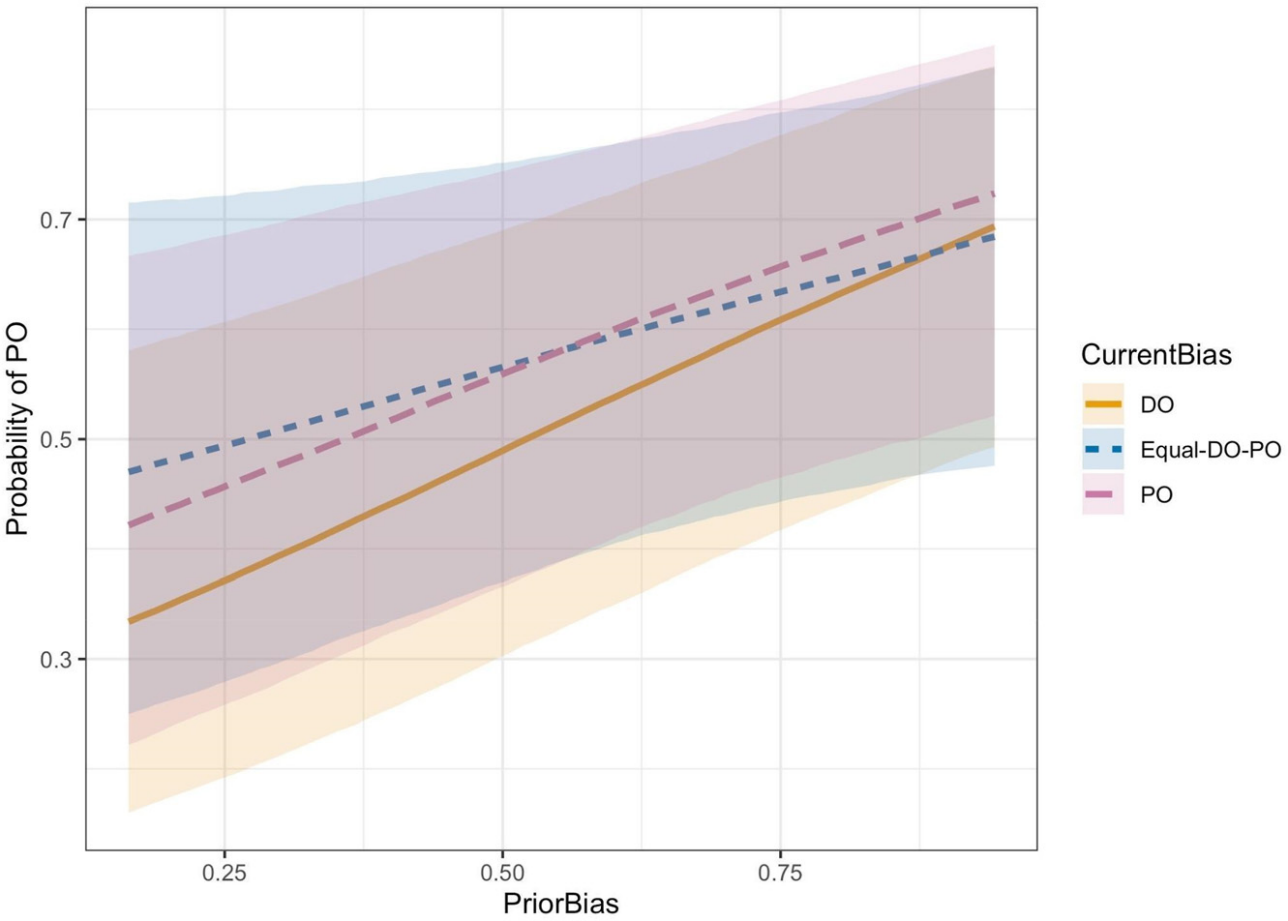
No convincing interaction between *CurrentBias* and *PriorBias* (towards PO). The DO bias and PO current bias lines are nearly parallel. If there were an interaction due to error-based learning, the DO bias line would be flatter than the PO bias line because hearing a DO structure should be more surprising and therefore more likely to minimize or neutralize any effect of prior bias.

Critically however, we did not find an interaction between prior and current bias, which would reflect error-based learning. The model with the interaction term did not perform better than the more parsimonious model with respect to WAIC. When included, the interaction term for PO-only (compared to DO-only), which we were primarily interested in, was close to 0, with a mean posterior log-odds ratio of −0.30, 95 % credible interval between −1.11 and 0.50, and posterior probability of 22.5 %. Figure 3, which displays the probabilities of PO predicted by the model with the interaction term, illustrates the lack of interaction. The PO and DO current bias lines are nearly parallel. If there were error-based learning, we would have expected the DO current bias line to have a shallower slope than the PO current bias line. The PO current bias line serves as a reference for slope because of the general prevalence of PO in English (i.e., hearing PO is not surprising). Thus, this line comes closest to reflecting the pure effect of prior bias alone, with low PO probability towards the left end of the line (strongly DO-biased verbs) and ceiling PO probability towards the right end (strongly PO-biased verbs). In comparison, the DO current bias line should show strong effects of learning within the experiment because DO is less common. Hearing a strongly PO-biased verb in DO (right end) should be very surprising and lead to a large adjustment, increasing probability of DO and decreasing probability of PO such that it comes close to the PO probability at the left end, resulting in a shallow slope. As can be seen in Figure 3, we did not see this pattern.

We did not have a strong prediction for the interaction between Equal-DO-PO (vs. DO-only) and prior bias because it is unclear what the surprisal effects would be for verbs that were heard in both DO and PO within the experiment. Therefore, we report this effect for completeness only. The mean posterior log odds-ratio was −0.80. The 95 % credible interval was between −1.75 and 0.16. The posterior probability of log odds-ratio being above zero was 5.45 %.

## 3 Testing the semantic similarity prediction

The results of the Bayesian analysis did not conform to the predictions of error-based learning. Although both prior and current bias led to the expected effects on construction choice during language production, we did not find evidence for an interaction between the two variables. In secondary analyses, we tested a prediction from exemplar theory, namely that semantic similarity between verbs would influence the choice between DO and PO.

### 3.1 Methods

We computed semantic similarity between verbs using the word2vec package in R (Wijffels 2020). The algorithm was trained on a subset of COCA (approximately 2.5 M words from each of the corpus subcomponents). Every token in the training corpus was presented as a triplet of wordform, lemma, and part of speech (e.g., *makes/make/vvz*) in order to disambiguate between verb and non-verb uses. Some of the verbs, such as *show* and *roll*, frequently occur as nouns, and it was important to exclude those irrelevant uses. No information about syntactic functions or subcategorization frames was explicitly used. In order to create the language model, we used 50 semantic dimensions and the continuous bag-of-words method, which works fast and is particularly well suited for frequent words (Mikolov et al. 2013). For each of the ten verbs, all inflected forms with verb tags (e.g., give/vv0, give/vvi, gives/vvz, gave/vvd, given/vvn and giving/vvg) were conflated into one string (e.g., give_VERB for *give*). This was done with the purpose of obtaining one semantic vector for every verb, regardless of its morphological form.

Next, we computed the similarity scores between all pairs of verb vectors as the square root of the average inner product of the vector elements capped to zero. We normalized the scores using the following procedure. First, we subtracted the minimum similarity value that occurred in the matrix from each score. Then, we divided each resulting score by the difference between the maximum similarity score (1) and the minimum value. Thus, the normalized similarity scores were in the range from 0 to 1 instead of the original smaller range from 0.58 to 0.92 (see Appendix). The scores correspond well with linguistic intuitions. For example, verbs of specific caused motion (*toss*, *throw*, *slide* and *roll*) have high similarity with each other, and the verb *show*, which represents metaphoric transfer of information, is similar to *give* and not much else.

Having computed each verb’s normalized semantic similarity to each other verb, we then computed a measure of how similar a given verb was to DO-only versus PO-only verbs in a given counterbalanced list within the experiment. This DO-only similarity/PO-only similarity measure is the ratio of the sum of similarity of the verb to all DO-only verbs (except for the verb itself, if the verb was in the DO-only condition in that list) to the similarity of the verb of all PO-only verbs (except for the verb itself, if the verb was in the PO-only condition in that list). See Table 3. A number greater than one indicates that the verb was semantically more similar to verbs that appeared in the DO-only condition than to verbs in the PO-only condition. A number less than one indicates the reverse, i.e., greater similarity to PO-only than DO-only verbs. The range of values for each verb across different lists reflects the random assignment design i.e., a verb’s semantic neighbors happened to be distributed more in the DO-only condition in some lists and more in the PO-only condition in others.

**Table 3:** DO/PO similarity scores per list and verb.

Verb	List 1	List 2	List 3	List 4	List 5
toss	1.1989	0.901864	0.542638	0.656981	2.757232
pass	1.14733	0.949111	0.765545	1.096114	1.119066
Bring	0.978708	1.184169	0.81802	0.92386	1.187836
throw	0.844489	1.473577	0.676049	0.725437	1.609392
Show	1.321874	1.256164	0.441124	1.045152	1.182858
Give	1.116721	0.990984	0.837278	0.68156	1.512129
Roll	1.202277	0.998942	0.593801	0.721968	2.108882
Mail	1.17492	0.51496	1.232524	1.168992	0.994087
Offer	0.922061	0.877024	1.020949	1.422273	0.776972
Slide	0.737891	2.297245	0.72343	0.213603	2.586136

The numbers from Table 3 were entered into the model under a variable named *Verb_DO_PO_sim*. As before, interactions were accepted if their 95 % credible intervals did not include zero (no new interactions met this criterion). The goodness of fit of the new model was comparable to the previous model: the average Bayesian C-index was 0.849 (95 % CI 0.842–0.855). The predictive power according to the WAIC criterion was not better. In addition, we also fitted a model excluding all observations with the Equal-DO-PO verbs. It performed somewhat better: the C-index was 0.852 (95 % CI 0.843–0.859).

### 3.2 Results

**Figure 4:**
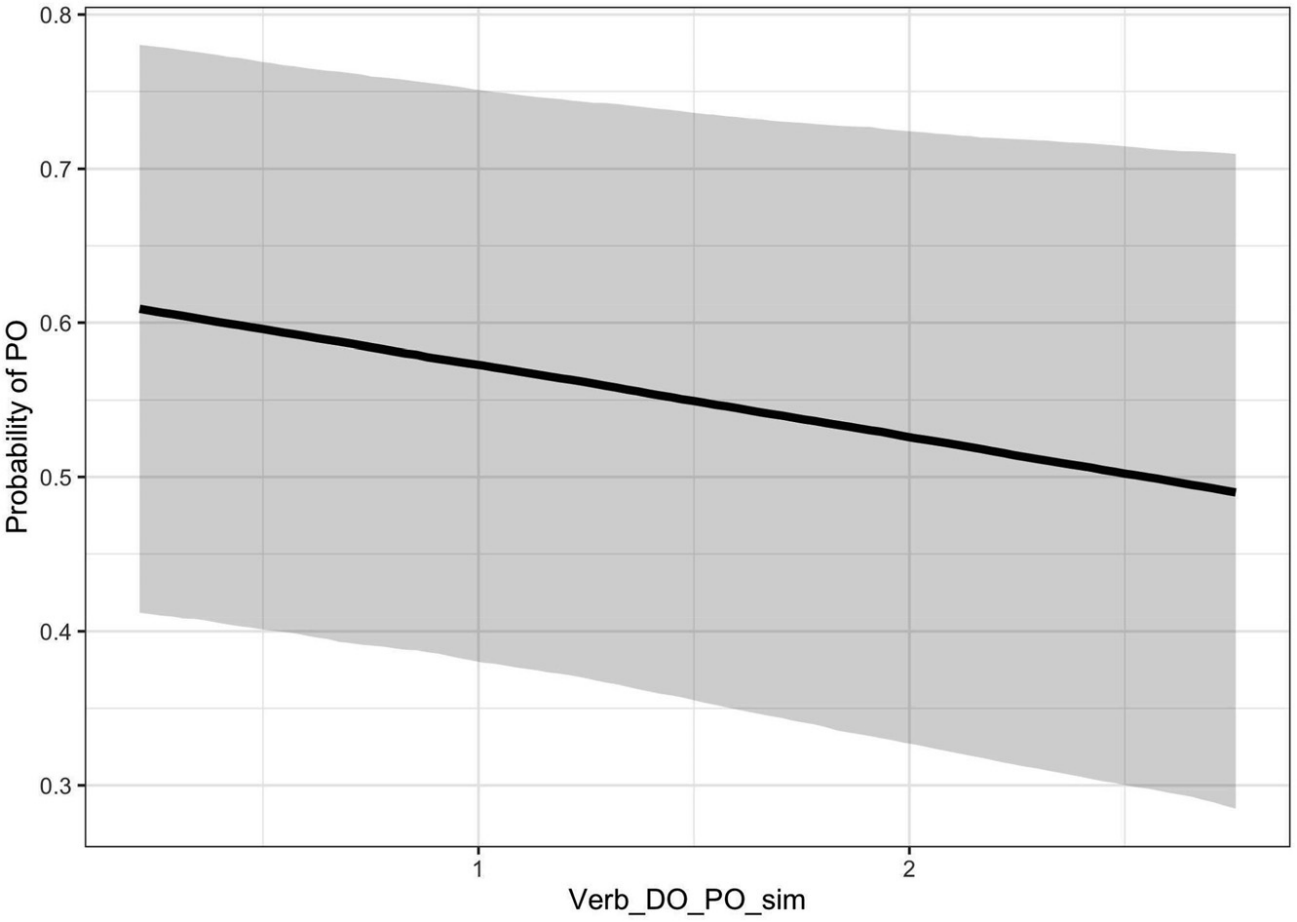
The negative effect of *Verb_DO_PO_sim* (similarity to DO-only vs. PO-only verbs) on the probability of producing PO, according to the model with all data points. If a verb was more semantically similar to DO bias than PO bias verbs, it had a lower probability of PO (corresponding to higher probability of DO).

The fixed-effects estimates based on the full dataset are displayed in Table 4, whereas Table 5 shows the estimates based on the dataset without the Equal-DO-PO verbs. In both cases, the estimate of *Verb_DO_PO_sim* had a negative value. This means that if a verb was more similar on average to the DO-only verbs in a List than to the PO-only verbs in the same List, then DO was more likely to be used and PO was less likely to be used. The effect is shown in Figure 4.

**Table 4:** Fixed-effects coefficients in the model including verb similarity: full dataset with all verbs (3,035 observations).

Regression term	Mean posterior (log-odds)	Lower 95 % CI	Upper 95 % CI	Posterior probability *b* > 0
Intercept	−0.91	−2.05	0.30	6.27 %
Trial	0.02	−0.01	0.06	90.76 %
Block = 2 (vs. 1)	−0.20	−0.60	0.19	15.58 %
PreviousResponse = PO	0.67	0.15	1.21	99.43 %
CurrentBias = Equal-DO-PO (vs. DO-only)	0.20	−0.06	0.46	93.84 %
CurrentBias = PO-only (vs. DO-only)	0.24	0.04	0.46	98.84 %
PriorBias in favor of PO	1.67	0.52	2.73	99.53 %
**Verb_DO_PO_sim**	**−0.19**	**−0.42**	**0.04**	**6.48 %**
ObjectLonger = Yes (vs. No)	0.09	−0.15	0.32	76.78 %
Trial:PreviousResponse = PO (vs. DO)	−0.05	−0.08	−0.01	0.91 %

The relevant new predictor (verb similarity) is highlighted in bold.

**Table 5:** Fixed-effects coefficients in the model including verb similarity: dataset without Equal-DO-PO verbs (2,408 observations).

Regression term	Mean posterior (log-odds)	Lower 95 % CI	Upper 95 % CI	Posterior probability *b* > 0
Intercept	−0.81	−2.02	0.41	9.04 %
Trial	0.01	−0.03	0.05	73.05 %
Block = 2 (vs. 1)	−0.16	−0.55	0.21	16.76 %
PreviousResponse = PO	0.65	0.09	1.24	98.89 %
CurrentBias = PO-only (vs. DO-only)	0.24	0.03	0.45	98.6 %
PriorBias in favor of PO	1.80	0.56	2.99	99.44 %
**Verb_DO_PO_sim**	**−0.24**	**−0.50**	**0.03**	**3.93 %**
ObjectLonger = Yes (vs. No)	−0.01	−0.27	0.25	45.66 %
Trial:PreviousResponse = PO (vs. DO)	−0.04	−0.08	0	2.8 %

The relevant new predictor (verb similarity) is highlighted in bold.

The effect was slightly stronger when the Equal-DO-PO verbs were excluded (Table 5). These findings are in accordance with our hypothesis. Note that the 95 % credible intervals include zeros, but only marginally. We have in both cases a higher than 90 % posterior probability that the similarity measure has a negative effect on the chances of PO. Overall, these results are not 100 % conclusive but the evidence weighs in favour of our hypothesis (cf. Vasishth et al. 2013). This evidence can be used later for identification of potentially relevant variables for future studies of the dative alternation. Detection of such suggestive effects is one of the epistemological advantages of using Bayesian models (cf. Levshina 2022).

## 4 General discussion

We investigated the potential mechanisms underlying the adaptation of sentence production to ongoing input. Bayesian analysis of adults’ dative sentence production showed that speakers’ choice of DO versus PO structure was influenced both by the verb’s prior bias, and the verb’s current bias within the context of the experiment. However, there was no interaction between prior and current bias, contrary to the prediction from error-based learning. Additional analysis suggested that semantic similarity between verbs influenced the likelihood of producing DO or PO, consistent with exemplar-based models of language. These results provide insight into and raise questions about the mechanisms used for updating language.

The present results add to the conclusions in Thothathiri et al. (2017) regarding the malleability of the language system. Even short exposures during an experiment appear to be sufficient to trigger learning and updating, in second language learners as well as native speakers (Gries 2019; Ryskin et al. 2017; Thothathiri and Braiuca 2021). Verb bias manipulations within the experiment have a detectable effect on subsequent language use even when accounting for prior verb bias accumulated through lifelong experience. This is especially noteworthy when the stimuli are in the participants’ native language because it suggests that comprehension and production are agile in adapting to new linguistic contexts. Based on a reviewer’s request, we tested a dichotomous low versus high prior frequency in datives overall variable—it did not improve the model (see Supplementary Material SM3). Looking at just low-frequency versus just high-frequency verbs, the CurrentBias coefficients pointed in the same direction for both. The effects were numerically smaller for low-frequency than high-frequency verbs. The 95 % credible interval included zero for the former but not the latter. Thus, the results suggested that, if anything, verbs with higher prior frequency may have shown better adaptation to the current bias, reinforcing our conclusion about flexibility in the language system.

The integration of past and new experiences is a key component of error-based theories of language learning, which propose that new input that is surprising from the perspective of past experience should have a stronger effect than input that is consistent with past experience (and is therefore less surprising). Thus, we would have expected an interaction between prior and current verb bias wherein verbs with a strong prior bias should show a larger effect of current (experimental) bias compared to verbs that have a weak prior bias especially when they occur in the less common DO structure. We did not find such an interaction. These results contrast with previous findings (Bernolet and Hartsuiker 2010; Lin and Fisher 2017). Bernolet and Hartsuiker (2010) found surprisal effects in syntactic priming i.e., in the effect of a prime sentence on an immediately following target sentence. Lin and Fisher (2017) reported longer-term surprisal effects in a training paradigm that is more akin to what was tested here. However, there were several methodological differences between that and the present study. Importantly, in Lin and Fisher’s study, each participant was in a current bias consistent with prior bias (DO-biased trained in DO, PO-biased trained in PO) or a current bias inconsistent with prior bias (DO-biased trained in PO, PO-biased trained in DO) condition. Thus, whether a participant experienced surprising verb-structure combinations was manipulated between subjects. Additionally, each participant was trained and tested with only two critical verbs. In contrast, in the present study, any given participant experienced a more complex combination of current and prior biases, with some verbs appearing in expected structures (consistent with prior bias) and others in surprising structures (inconsistent with prior bias). Further, we used 10 verbs with prior bias represented along a spectrum instead of dichotomously as DO- or PO-biased. Therefore, it remains to be seen whether long-term surprisal effects can be detected replicably in a within-subjects design.

The fact that we found additive rather than interactive effects of prior bias and current bias suggests that some other mechanism other than error-based learning was operational in the context of the present study. Speakers were sensitive to both prior and current bias, indicating that the learning mechanism was sensitive to the statistics of linguistic experience. Broadly speaking, exemplar-based models emphasize the role of repetition in strengthening linguistic representations (e.g., Bybee 1985, 2013) and can therefore capture such sensitivity to statistics. Statistical experience can be conceptualized simply as relative frequency or conditional probability P(Construction|Verb) and there is some evidence in the literature that simple conditional probabilities perform at least as well as more sophisticated measures in predicting different kinds of linguistic behaviour (Divjak 2019: Section 2.2, 152–153). More narrowly, the current results favor the sub-category of exemplar-based models that presuppose that learning depends on tracking usage statistics but do not require learning to depend on prediction and error. Conversely, these findings do not support models that argue for error-based learning as the primary mechanism for updating verb-structure links in all contexts.

Which learning mechanism is most useful for updating verb-structure statistical associations could vary depending on context. Language use varies between individual speakers, communities and communicative situations (e.g., registers, genres and text types). Pragmatic associations of linguistic forms with co-textual and contextual properties, for instance, are entrenched in individual minds and conventionalized in the speech community, alongside symbolic, paradigmatic and syntagmatic associations (Schmid 2020). Labov’s (1972) famous distinction between indicators, markers and stereotypes suggests that the strength of these associations represents a continuum. Statistical information gathered from one particular social context may be useful to generalize broadly in some cases (e.g., second language learning in a classroom) but might be more narrowly applicable in others (e.g., morphological and syntactic patterns signifying sociolinguistic distinctions). Predictions from prior experience and adjustments to the language system based on deviations from that prediction might be more useful in the former than the latter case. The present study tested learning and production in a particular context with distinctive visual and linguistic features (hand transferring an object to a puppet animal in different ways against the same background and sentences that all began with “Kate gave”). Similar to a social context containing particular usage patterns that language users might not need to generalize outside of that context, this could have favored a context-specific learning mechanism over prediction-and-error-based learning. Tracking and using context-specific statistical information is rational (Ellis 2006), and empirical evidence suggests that the human brain is capable of doing so (e.g., Kamide 2012; Kroczek and Gunter 2017). Future studies could manipulate the extent of similarity between training and test stimuli as well as the distinctiveness of the overall experimental context to investigate whether language updating under different conditions uses different mechanisms.

Attention could be another factor that influences when error-based learning is used. While tracking and using probabilities can happen with or without attention (e.g., see Duncan and Theeuwes 2020 and references therein), using prediction error appropriately might require the use of attention to ensure that we do not update our model of the world based on unreliable information (den Ouden et al. 2012). In the present case, the participants’ brains had clearly tracked the probabilities of a verb being used in DO and PO (implicitly), as shown by their learning the biases within the experiment. However, it is possible that participants’ attention was drawn more to the visual aspects of the experiment (e.g., puppets) instead of the linguistic setting, which could have decreased the likelihood of using linguistic prediction errors and error-based learning (den Ouden et al. 2012). Future studies that manipulate where attention is directed can shed light on whether error-based learning is used particularly when the linguistic and communicative setting is made more salient than non-linguistic aspects.

In secondary analyses, we found suggestive effects of semantic similarity. Exemplar-based models of language propose that the language system stores exemplars of linguistic representations along with their type and token frequency. Representations can span the range from item-specific constructions to more abstract constructions based on semantic (and other kinds of) similarity. Beyond allowing for frequency-tracking effects (e.g., verb bias), this class of models postulate that the similarity of a verb to other verbs can influence production. Thus, how often verb X is used in DO versus PO depends not only on how often X was experienced in DO versus PO but also on the biases of the other verbs that are semantically similar to X. While previous studies have demonstrated an effect of semantic similarity on language use (Bybee and Eddington 2006; Yi et al. 2019), we show here that this effect extends to statistical information acquired recently from new language input. Specifically, *semantically similar verbs’ biases within the context of the experiment* influenced speakers’ choice of DO versus PO with a particular verb. Thus, the results suggest that speakers can track and use new biases acquired within a short exposure session, and that semantically organized verb categories learned via lifelong experience can be integrated with the new biases. Context-specific analogies could possibly support such integration. As discussed above, the present study contained recognizably similar visual and linguistic features across trials and verbs. This could have encouraged analogizing from one verb to another, modulated by the semantic similarity between those verbs. It should also be noted that the semantic similarity findings were tentative rather than strong. It is possible therefore that the core results, i.e., an effect of prior and current bias, could be explained by statistical learning alone without recourse to predictions, exemplars, or analogies.

The paradigm used in the present study allowed for a systematic way to manipulate variables of interest (e.g., verb bias) while minimizing the influence of extraneous variables (e.g., visual or attentional differences between events). In this sense, the experimental setting was similar to structured interactions in real life where the communicative context is more or less predictable (e.g., conference talks at a podium using the same screen). The paradigm can be extended to include more variation in sentence structures, speakers, and visual settings in order to mimic less structured communicative contexts. This can help clarify whether the present findings selectively apply to structured contexts only or whether they are more broadly generalizable to more varied contexts. Additionally, future studies where context is a fixed variable of interest can shed light on whether the mechanisms used to update usage statistics (non-error-based vs. error-based) change systematically according to how structured or unstructured the context is.

In conclusion, we found evidence that speakers can update verb biases dynamically based on new language experiences manipulated during a short experimental training session. At the same time, prior language experience also influenced speech. Further, semantic similarity based on past experience influenced how verb biases manipulated within the experiment were extended across verbs. Thus, there was some integration between past and current experience. But this integration did not show the signature pattern expected from error-based learning. Future studies are needed to fully understand the situations under which language users use error-based learning, and update their native languages either broadly or in more context-specific ways.

## Data availability statement

Data and code for this study is available at https://osf.io/3ca8p.

## Supplementary Material

Supplementary Material

## Figures and Tables

**Table:** 

	Toss	Pass	Bring	Throw	Show	Give	Roll	Mail	Offer	Slide
Toss	1	0.51	0.53	0.71	0.13	0.3	0.6	0.25	0.09	0.64
Pass	0.51	1	0.7	0.47	0.41	0.61	0.54	0.42	0.51	0.54
Bring	0.53	0.7	1	0.61	0.49	0.75	0.7	0.4	0.58	0.59
Throw	0.71	0.47	0.61	1	0.28	0.52	0.69	0.13	0.12	0.64
Show	0.13	0.41	0.49	0.28	1	0.68	0.25	0.02	0.38	0.23
Give	0.3	0.61	0.75	0.52	0.68	1	0.43	0.16	0.68	0.3
Roll	0.6	0.54	0.7	0.69	0.25	0.43	1	0.17	0.03	0.81
Mail	0.25	0.42	0.4	0.13	0.02	0.16	0.17	1	0.43	0.06
Offer	0.09	0.51	0.58	0.12	0.38	0.68	0.03	0.43	1	0
Slide	0.64	0.54	0.59	0.64	0.23	0.3	0.81	0.06	0	1
