# Supplementary material for: Updating constructions: additive effects of prior and current experience during sentence production
Source: Cogn Linguist. 2023 Oct 3;34(3-4):479–502. doi: 10.1515/cog-2022-0020 (PMC10630066; doi:10.1515/cog-2022-0020)
Supplement: Supplementary file 1 — Supplementary Material [file j_cog-2022-0020_suppl_001.docx]

**Supplementary Materials (SM)**

**SM1. Testing log-odds instead of proportions of PO for PreviousBias**

We also tested log-odds of PO instead of proportions of PO as PreviousBias. The log-odds are very correlated with the proportions, as the plot below shows. Pearson’s correlation coefficient is 0.987 (p < 0.0001).


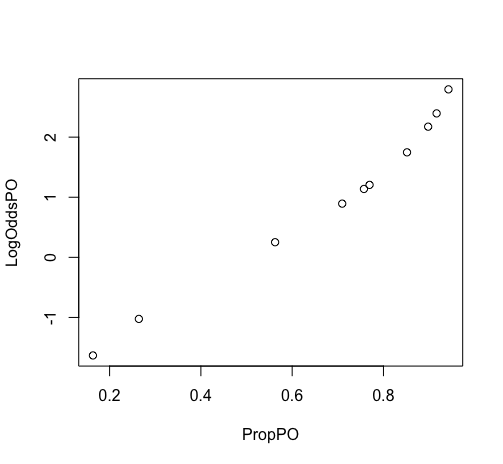


The model with log-odds does not perform better than the model with proportions. The elpd_diff stands for the difference between the expected log predicted density scores of the models. ELPD is a measure of accuracy. The negative difference between the old and new models means that the old model is better. The difference is also small given the error (se_diff). As a very informal rule of thumb, elpd_diff should be 2-5 times as large as se_diff, for one model to be preferred over the other. This is not the case here, so we used the model with proportions because proportions are easier to interpret.

> loo_compare(brm1, brm4, criterion="waic")

elpd_diff se_diff

brm1 0.0 0.0

brm4 -4.6 37.8

The log-odds of PO estimates: 0.34, 95% credible interval [0.14, 0.54].

The PO proportion estimates: 1.65, 95% credible interval [0.55, 2.65].

The other estimates (except for the intercept) are very close.

**SM2. Testing a random slope for the Trial*PreviousResponse interaction**

We tested a random slope for the interaction between *Trial* and *PreviousResponse*. Our WAIC model comparison does not show evidence in favor of adding this random slope. The model brm1 without the random slope is better than the model brm3 with the random slope.

> loo_compare(brm1, brm3, criterion="waic")

elpd_diff se_diff

brm1 0.0 0.0

brm3 -3.3 1.0

**SM3. Testing the effect of frequency and comparing effects for low-frequency versus high-frequency verbs**

Based on a reviewer’s request, we checked whether a dichotomous low vs. high frequency factor influenced the results. We also compared the results for just low-frequency versus just high-frequency verbs to see if there was a qualitative difference.

The table below shows the frequency of the 10 dative verbs in dative constructions. We classified the top 5 verbs as high frequency (*bring, give, offer, pass* and *show*) and the bottom 5 as low frequency (*mail, roll, slide, toss* and *throw*).

| **Verb** | **Total frequency in both datives** |
| --- | --- |
| give | 165,254 |
| bring | 36,223 |
| show | 15,367 |
| offer | 9,783 |
| pass | 3,360 |
| throw | 2,180 |
| toss | 828 |
| mail | 512 |
| roll | 395 |
| slide | 191 |

The model did not improve when we included the dichotomous variable VerbFreq. According to the WAIC model comparison, the model with VerbFreq is slightly worse than the model without VerbFreq. Thus, we did not find evidence in favor of including this variable.

> loo_compare(brm1, brm2, criterion="waic")

elpd_diff se_diff

brm1 0.0 0.0

brm2 -0.2 1.3

The same results were obtained for introducing the interactions between VerbFreq and the other predictors. In none of them did we find support for including this variable and its interaction term.

Comparing the results for low-frequency verbs only to that for high-frequency verbs only, we found that the coefficients pointed in the same direction. The effect of CurrentBiasPO (vs DO) was numerically lower for low-frequency than high-frequency verbs. The 95% credible interval included zero for the former but not the latter.

*Results for Low-Frequency Verbs*

Estimate Est.Error l-95% CI u-95% CI

Intercept -1.12 1.53 -3.87 1.89

Trial 0.01 0.03 -0.03 0.07

Block -0.21 0.24 -0.68 0.25

PreviousResponsePO 1.21 0.43 0.38 2.04

CurrentBiasEqui 0.15 0.21 -0.26 0.54

**CurrentBiasPO 0.09 0.16 -0.22 0.42**

ObjectLongerYes -0.10 0.20 -0.52 0.30

PreviousBias 2.06 1.73 -1.26 5.24

Trial:PreviousResponsePO -0.03 0.03 -0.09 0.03

*Results for high-frequency verbs*

Estimate Est.Error l-95% CI u-95% CI

Intercept -0.90 0.82 -2.48 0.75

Trial 0.03 0.02 -0.02 0.08

Block -0.17 0.26 -0.70 0.35

PreviousResponsePO 0.75 0.36 0.03 1.48

CurrentBiasEqui 0.23 0.19 -0.13 0.60

**CurrentBiasPO 0.38 0.15 0.09 0.68**

ObjectLongerYes 0.05 0.22 -0.39 0.47

PreviousBias 0.98 0.79 -0.67 2.47

Trial:PreviousResponsePO -0.05 0.03 -0.11 0.00
